# Capsaicin Induces G2/M Cell-Cycle Arrest and Impairs Autophagy in Caco-2 Cells: An Exploratory Study

**DOI:** 10.3390/ijms27198435

**Published:** 2026-09-22

**Authors:** Hla Sudan, Sofia Passaponti, Roberta Romagnoli, Laura Cresti, Mariangela Gentile, Maria Frosini, Anna Maria Aloisi

**Affiliations:** 1 Department of Medicine, Surgery and Neuroscience, University of Siena, 53100 Siena, Italy; sofia.passaponti2@unisi.it; 2 Department of Life Sciences, University of Siena, 53100 Siena, Italy; roberta.romagnoli@unisi.it (R.R.); laura.cresti@unisi.it (L.C.); mariangela.gentile@unisi.it (M.G.); maria.frosini@unisi.it (M.F.)

**Keywords:** capsaicin, autophagy, colon cancer, migration behavior, lysosomes

## Abstract

Capsaicin, the principal bioactive compound of chilli peppers, has emerged as a promising natural molecule with anticancer potential in several malignancies, including colorectal cancer. However, the molecular mechanisms underlying its effects in colorectal cancer cells remain incompletely understood. In the present study, we investigated the effects of capsaicin on Caco-2 human colorectal adenocarcinoma cells, focusing on cell viability, proliferation, migration, cell-cycle regulation, and autophagy. Capsaicin (50–200 µM) reduced cell viability in a dose- and time-dependent manner and significantly inhibited proliferation and migratory capacity. Capsaicin induced G2/M cell-cycle arrest, accompanied by increased p21 expression and reduced p53 phosphorylation. Notably, capsaicin-induced cytotoxicity was not associated with activation of caspase-dependent apoptosis and was instead associated with marked accumulation of autophagic structures and increased LC3B-II/LAMP-1 co-localization. Chloroquine co-treatment failed to further increase LC3B-II or p62 accumulation, indicating that autophagic degradation was already impaired. Although lysosomal abundance remained unchanged, capsaicin significantly reduced mature cathepsin-D protein, demonstrating compromised lysosomal degradative capacity and defective autophagic flux. In addition, capsaicin upregulated occludin expression, suggesting enhanced tight-junction integrity and reduced migratory potential. Collectively, these findings, at least explorative, demonstrate that capsaicin reduces Caco-2 cell viability and induces G2/M cell-cycle arrest, accompanied by p21 upregulation and impaired lysosomal degradative capacity, highlighting disruption of the autophagy–lysosomal pathway as a potential mechanism underlying its anticancer activity in Caco-2 cells.

## 1. Introduction

Autophagy is a conserved self-degradation pathway through which the cell recycles intracellular components to maintain metabolic and structural homeostasis. The process involves sequential steps, including autophagosome formation, fusion with lysosomes, and autolysosome maturation, where lysosomal enzymes degrade the sequestered cargo. The resulting metabolites can be reutilized to support energy production and biosynthesis, while autophagic degradation also facilitates the removal of damaged organelles and potentially harmful or toxic cellular constituents. Impairment at any stage of this pathway disrupts cellular homeostasis and compromises cell viability [1].

Multiple studies conducted in cancer cell lines have demonstrated that disruption of the autophagic pathway is associated with reduced cell viability, impaired proliferative capacity, and the activation of programmed cell death pathways, particularly apoptosis [2,3]. Conversely, excessive or uncontrolled activation of autophagy can itself trigger a distinct form of programmed cell death, commonly referred to as autophagic cell death (ACD) [4].

Colorectal cancer (CRC) arises in the colon or rectum, segments of the large intestine, and represents one of the most prevalent malignancies globally. It is the second leading cause of cancer-related mortality in the United States and constitutes the primary cause of cancer death among adults under 50 years of age [5]. Metastatic colorectal cancer remains a leading cause of cancer-related mortality worldwide. Despite advances in metastatic colorectal cancer therapy, the near-inevitable emergence of chemotherapy resistance underscores the urgent need for novel agents to augment current treatment regimens [6].

Accordingly, the discovery of bioactive compounds with robust anticancer activity has become a critical priority for overcoming therapeutic resistance and strengthening existing treatment options. Capsaicin (8-methyl-N-vanillyl-6-nonenamide) is the main pungent alkaloid found in chilli peppers of the Solanaceae family. It has been used for centuries in traditional medicine as an analgesic for several illnesses. It also exhibits anti-inflammatory, antioxidant, and antimicrobial activities [7]. Additionally, it has gained attention as a promising natural compound with antineoplastic activity. Different mechanisms of action have been identified to explain the anti-cancer activity of capsaicin across tumour types. For instance, it was reported that capsaicin, via the vanilloid receptors, primarily Transient Receptor Potential Vanilloid 1 (TRPV1), induces an imbalance in intracellular calcium homeostasis, resulting in apoptosis in glioma cells [8]. Moreover, capsaicin has been shown to downregulate the Phosphoinositide 3-Kinaseserine/Threonine-protein kinase/Mammalian Target Of Rapamycin (PI3K/AKT/mTOR) pathway in human nasopharyngeal carcinoma cells, thereby activating two forms of programmed cell death, autophagy and apoptosis [9]. In pheochromocytoma cells (PC12), capsaicin induces apoptosis primarily by activating the endoplasmic reticulum (ER) stress pathway and driving ER stress–dependent apoptotic signalling [10]. Additionally, capsaicin demonstrates significant anti-migratory activity in oral cancer cells [11]. In AsPC-1 and BxPC-3 pancreatic cancer cells, capsaicin induces apoptosis primarily through reactive oxygen species (ROS) generation, persistent mitochondrial membrane depolarization, and activation of the JNK signaling pathway. This process leads to Bax upregulation, Bcl-2 and survivin downregulation, and cytosolic release of cytochrome-c and apoptosis-inducing factor (AIF) [12]. Furthermore, Capsaicin inhibits breast cancer cell growth by inducing G0/G1 cell-cycle arrest and promoting apoptosis through downregulation of cyclin D1, alongside increased caspase activation and PARP cleavage. It also suppresses cell migration and reduces tumour burden in vivo, acting as a potent modulator of the EGFR/HER-2 signaling axis in breast cancer models [13].

In colon cancer, the mechanisms responsible for the anti-cancer effect of capsaicin are not yet completely understood. For instance, it is reported that capsaicin decreased the growth of HCT116 cells by mediating cell cycle (G0/G1 phase arrest) and inducing apoptosis [14]. In Colo320DM and LoVo cells, results showed that capsaicin-induced apoptosis is associated with an increase in ROS generation and a disruption of the mitochondrial transmembrane potential [14].

Capsaicin was selected for this study because colorectal cancer urgently requires new bioactive substances capable of overcoming chemotherapy resistance and targeting alternative survival pathways. Capsaicin is particularly relevant in this context, as it can activate different forms of programmed cell death, including apoptosis, autophagic cell death, and ER-stress-mediated apoptosis, and impair key survival mechanisms such as autophagy. Notably, its anticancer effects are highly cell-type dependent, with distinct mechanisms reported across different colorectal cancer models. The Caco-2 colon carcinoma cell line is widely used in intestinal-barrier and absorption studies because these cells spontaneously differentiate over approximately 14 days into a polarized monolayer exhibiting morphological and functional characteristics of absorptive enterocytes, including the formation of a brush-border membrane [15]. To ensure that the cells retained their malignant phenotype and did not undergo enterocyte-like differentiation, all experiments in the present study were conducted within a culture period not exceeding seven days. Under these conditions, Caco-2 cells serve as an appropriate model of colorectal cancer [16,17]. The anti-cancer effects of several chemotherapy drugs on Caco-2 cells are reported by previous studies. The study by Keramati Z et al. (2023) demonstrated that CeO_2_-XG nanoparticles markedly enhanced the anticancer activity of 5-fluorouracil in Caco-2 cells. Co-treatment reduced the IC_50_ of 5-FU, suppressed anti-apoptotic and chemokine-related gene expression, and upregulated key pro-apoptotic regulators, resulting in more than a two-fold increase in apoptotic cell death compared with 5-FU alone [18]. Furthermore, Oxaliplatin induces apoptotic cell death and activates a cytoprotective autophagy pathway driven by endoplasmic reticular (ER) stress and ROS in Caco-2 cells [19]. The impact of capsaicin on Caco-2 cells is not well defined. Caco-2 cells represent a widely accepted human in vitro model for the intestinal epithelium; however, according to the Human Protein Atlas, they are characterized by relatively low basal expression levels of the TRPV1 channel, one of the primary targets of capsaicin [20]. Selecting this model allows us to explore the cellular and molecular response to capsaicin under conditions where the canonical receptor expression is limited, helping to clarify whether its anticancer activity requires high local concentrations and could involve TRPV1- independent pathways. Accordingly, this study aimed to investigate the cellular and molecular effects of capsaicin in Caco-2 cells, with a focus on pathways related to cell viability, proliferation, autophagy, and cell-cycle regulation.

## 2. Results

### 2.1. Capsaicin Induces Dose- and Time-Dependent Cytotoxicity and Proliferation Inhibition in Caco-2 Cells

The cytotoxic effects of capsaicin (CAP) on Caco-2 cells were initially evaluated using the MTT assay across multiple concentrations and incubation periods. Cells were exposed to 50, 100, and 200 µM capsaicin for 24, 48, and 72 h, respectively (Figure 1a). Capsaicin elicited a pronounced dose- and time-dependent decline in cell viability, reaching 54.82 ± 2.90% at 200 µM after 72 h (*p* < 0.001; 95% CI: 47.3% to 58.7%, *d* = 12.72) (Figure 1b). Based on these results, 200 µM was selected for all subsequent experiments. The vehicle-treated group showed negligible cytotoxicity (2.0 ± 3.4%) compared with the untreated control, with no statistically significant difference (*p* > 0.05), confirming that the observed reduction in viability was attributable to capsaicin rather than the vehicle.

To verify the impact on proliferation, the SRB assay was conducted with absorbance measured at 0, 24, 48, and 72 h. CAP treatment resulted in significant time-dependent suppression of cell proliferation, reducing cell mass to approximately 62.28 ± 5.60% of control levels after 72 h (*p* < 0.05; 95% CI: 50.63% to 67.56%, *d = 5.50*) (Figure 1c). In contrast, the vehicle group exhibited only a minimal, non-significant reduction relative to the control (*p* > 0.05).

To further substantiate the antiproliferative activity of CAP, the wound closure assay was performed in the absence of a mitotic inhibitor. Scratch assay measurements were normalized to the corresponding SRB signal obtained under identical treatment conditions. Following 72 h of exposure to 200 µM CAP, normalized wound closure was markedly reduced (28.54 ± 14.38%) compared with the control group (100 ± 5.70%) (*p* < 0.05; 95% CI: 24.76% to 106.74%, *d = 3.7*) (Figure 1d). No significant difference was detected between the vehicle and control groups (*p* > 0.05). Because the assay was performed without an antiproliferative agent, the reduction in wound closure likely reflects a combined effect of impaired proliferation and diminished migratory capacity rather than a migration-specific response.

In line with these results, Trypan blue exclusion analysis demonstrated a marked increase in non-viable cells following capsaicin treatment, corresponding to an approximately four-fold elevation in cell death relative to the control group (*p* < 0.001; 95% CI: −3.49 to −2.13; *d* = 10.95) (Figure 1e). These data indicate that the reduction in SRB signal reflects both genuine cytotoxicity and impaired proliferative capacity.

### 2.2. CAP Induces G2/M Cell-Cycle Arrest Associated with p21 Upregulation and Reduced p53 Phosphorylation in Caco-2 Cells

Flow cytometric analysis of DNA content demonstrated clear shifts in cell-cycle distribution between control and CAP-treated Caco-2 cells (Figure 2). CAP exposure markedly decreased the Sub-G0/G1 population (1.73 ± 0.03% vs. 3.88 ± 0.33% in controls; *p* < 0.001; 95% CI: 1.10 to 3.19; *d* = 5.30), consistent with the absence of apoptotic DNA fragmentation. The proportion of cells in G0/G1 remained unchanged (*p* > 0.05). In contrast, CAP significantly reduced the S-phase fraction (12.70 ± 0.37% vs. 15.74 ± 0.32%; *p* < 0.001; 95% CI: 1.82 to 4.24; *d* = 5.07) and induced a pronounced accumulation of cells in the G2/M phase (29.19 ± 0.21% vs. 23.34 ± 0.22%; *p* < 0.001; 95% CI: −6.60 to −5.07; *d* = 15.70).

To further elucidate the molecular basis of this arrest, the expression of key cell-cycle regulators was examined. CAP treatment significantly upregulated the cyclin-dependent kinase inhibitor p21 (WAF1/Cip1) relative to the control group (*p* < 0.05; 95% CI: −3.29 to −2.81) (Figure 2e). In contrast, levels of phosphorylated p53 were markedly reduced following CAP exposure (*p* < 0.001; 95% CI: 0.94 to 1.04, *d* = 8.80), indicating that the canonical DNA-damage–p53 signalling axis was not activated (Figure 2f). The combined increase in p21 expression and reduction in phospho-p53 levels occurred in association with the observed G2/M cell-cycle arrest.

### 2.3. Absence of Caspase Activation and Nuclear Morphological Changes in CAP-Treated Caco-2 Cells

The potential contribution of caspase‑dependent apoptosis was examined by measuring the abundance of cleaved caspase‑3—an essential effector in both intrinsic and extrinsic apoptotic signaling—and by determining the ratio of cleaved to total poly(ADP‑ribose) polymerase (PARP), a widely used indicator of programmed cell death. In our analyses (Figure 3a), the cleaved caspase‑3/caspase‑3 ratio showed no significant difference between CAP‑treated cells and untreated controls (*p* > 0.05). A comparable pattern was observed for PARP, as the cleaved‑PARP/total‑PARP ratio remained unchanged following CAP exposure (Figure 3b). These biochemical data collectively suggest that CAP does not trigger caspase‑mediated apoptotic processes under the experimental conditions applied.Morphological assessment using DAPI staining further supported this conclusion. No nuclear abnormalities were detected in either control or CAP‑treated cells (Figure 3c). At 10× magnification, nuclei displayed uniform distribution and similar density across groups. Higher‑magnification imaging (63×) confirmed that nuclear structures were intact, round, and exhibited consistent DAPI fluorescence, with no signs of chromatin condensation, fragmentation, or other morphological features typically associated with apoptosis‑related nuclear damage.Overall, these findings indicate that capsaicin treatment does not elicit detectable alterations in nuclear morphology or activate caspase‑dependent apoptotic pathways in the examined model.

Morphological assessment using DAPI staining further supported this conclusion. No nuclear abnormalities were detected in either control or CAP-treated cells (Figure 3c). At 10× magnification, nuclei displayed uniform distribution and similar density across groups. Higher-magnification imaging (63×) confirmed that nuclear structures were intact, round, and exhibited consistent DAPI fluorescence, with no signs of chromatin condensation, fragmentation, or other morphological features typically associated with apoptosis-related nuclear damage.

Overall, these findings indicate that capsaicin treatment does not elicit detectable alterations in nuclear morphology or activate caspase-dependent apoptotic pathways in the examined model.

### 2.4. CAP Induces Impaired Autophagic Degradation

The autophagosome formation was quantified by analysing the conversion of cytosolic Microtubule-associated protein 1 light chain 3B (LC3B)-I to its lipidated form, LC3B-II. To validate the responsiveness of our model to autophagy induction, cells were subjected to serum starvation for 6 h prior to terminating the treatment. As shown in Figure 4a,b, starvation could not markedly increase LC3B-II levels and p62/SQSTM1 relative to control cells, which could be explained by efficient degradation in lysosomes (*p* > 0.05). To further assess autophagic flux, starved cells were co-treated with 50 µM and 75 µM of chloroquine (CQ), a lysosomal acidification inhibitor that typically promotes LC3B-II and p62 accumulation when autophagic degradation is functional. As expected, 75 µM of CQ treatment resulted in robust accumulation of both LC3B-II (*p* < 0.01; 95% CI: 1.12 to 2.5, *d* = 2.84) and p62/SQSTM1 (*p* < 0.01; 95% CI: 1.23 to 3.41, *d* = 1.29), confirming that this concentration effectively blocked lysosomal degradation and was suitable for subsequent flux assessment in CAP-treated cells. Moreover, 50 µM of CQ treatment resulted in an accumulation of LC3B-II (*p* < 0.01; 95% CI: 0.91 to 2.45, *d* = 3.65) without p62/SQSTM1 (*p* > 0.05). This pattern could be explained by incomplete blockade of autophagic degradation at this lower concentration, and/or a lower sensitivity of p62 to partial lysosomal inhibition compared with LC3B-II.

In CAP-treated cells, the LC3B-II/LC3B-I ratio was significantly elevated compared with the control group (*p* < 0.01; 95% CI: 2.47 to 5.41, *d* = 7.46), as presented in Figure 4c.

This elevation can reflect either an increase in autophagosome formation or a blockade in their lysosomal turnover. To differentiate between these possibilities, we examined markers of lysosomal structure and function. Lysosomal-associated membrane protein 1 (LAMP-1), a membrane protein commonly used to assess lysosomal abundance, showed no significant change in capsaicin-treated cells compared with controls (*p* > 0.05) (Figure 4d), indicating that lysosomal biogenesis or membrane content was not altered. In contrast, capsaicin markedly reduced the expression of the lysosomal protease mature cathepsin-D (*p* < 0.001; 95% CI: 0.615–1.35, *d* = 7.31), suggesting that lysosomal degradative capacity was compromised rather than lysosome number (Figure 4e), while there was no difference in pro-cathepsin D protein levels compared to the control group (Figure 4d) (*p* > 0.05).

To further assess autophagic flux, CAP-treated cells were co-treated with CQ. CQ failed to produce any additional increase in LC3B-II or p62 levels at either 50 or 75 µM (*p* > 0.05) (Figure 5a,b). The absence of CQ-induced accumulation indicates that autophagic degradation was already impaired before CQ exposure. Confocal microscopy further demonstrated that capsaicin markedly increased autophagosome–lysosome interactions. This was evidenced by a significant rise in LC3B-II/LAMP-1 co-localization in capsaicin-treated cells (Mander’s M1, auto-thresholding = 0.485 ± 0.02) compared with the control group (Mander’s M1 = 0.206 ± 0.03) (*p* < 0.001; 95% CI: 0.179–0.377) (Figure 5c).

In parallel, transmission electron microscopy revealed a pronounced accumulation of autophagic structures in capsaicin-treated cells relative to untreated controls, consistent with enhanced autophagosome abundance at the ultrastructural level. Capsaicin-treated cells exhibited a marked accumulation of double-membrane autophagosomes, whereas control cells displayed only occasional basal autophagic vacuoles (*p* < 0.01; 95% CI: 0.112–0.243). This ultrastructural increase reflects enhanced autophagosome formation and/or impaired autophagosome clearance, consistent with the lysosomal dysfunction indicated by cathepsin-D downregulation (Figure 5d).

To examine whether lysosomal proteolytic capacity was linked to cellular survival, the relationship between cathepsin-D protein expression and cell viability was quantified. A strong positive correlation was detected (Pearson r = 0.99, *p* < 0.01), indicating that higher cathepsin-D levels were associated with increased viability (Figure 6a). Conversely, reduced cathepsin-D expression correlated with elevated LC3B-II (Pearson r = −0.96, *p* < 0.01) and p62 levels (Pearson r = −0.91, *p* < 0.01), consistent with impaired autophagic degradation and a late-stage autophagy blockade driven by lysosomal dysfunction (Figure 6b,c).

### 2.5. CAP Upregulates Occludin Expression and Decreases the Migration Rate of Caco-2 Cells

To distinguish the anti-migratory effect of CAP from its antiproliferative activity, ara-C was used to suppress cell division in both control and CAP-treated groups. Ara-C alone did not alter migration (*p* > 0.05), confirming that proliferation was effectively blocked without affecting motility. Under these conditions, CAP reduced migration to 0.22-fold relative to ara-C controls (*p* < 0.01; 95% CI: 0.16 to 1.36, *d* = 4.37) (Figure 7a). Consistent with reduced motility, CAP also increased occludin expression (*p* < 0.01; 95% CI: 0.96 to 2.45, *d* = 7.51), supporting enhanced tight-junction integrity as a contributor to impaired migration (Figure 7b).

## 3. Discussion

This study provides preliminary evidence that capsaicin exerts cytotoxic and anti-proliferative effects in Caco-2 colorectal cancer cells through mechanisms involving cell-cycle arrest, lysosomal dysfunction, and impaired autophagic degradation. Specifically, capsaicin reduced cell viability, proliferation, and migratory capacity, while inducing a p21-associated G2/M arrest without activating caspase-dependent apoptosis. Capsaicin promoted autophagosome accumulation together with reduced cathepsin-D expression and impaired autophagic flux, indicating a late-stage blockade of autophagy associated with lysosomal dysfunction. In parallel, capsaicin increased occludin expression, suggesting additional effects on epithelial barrier organization and cell motility. Taken together, these exploratory findings identify lysosomal impairment and defective autophagic degradation as central events underlying the cytotoxic activity of capsaicin in Caco-2 cells.

Capsaicin, the most prominent bioactive ingredient of chilli peppers, has been used in traditional medicine for centuries worldwide to manage the chronic pain associated with a spectrum of diseases such as arthritis, diabetic neuropathy, and postherpetic neuralgia [21]. For that, capsaicin has been approved by the US Food and Drug Administration (FDA) as an 8% dermal analgesic patch to control neuropathic pain [22]. In addition to its antioxidant activity and antiulcer effects [23,24], capsaicin has demonstrated anticancer activity by targeting several genes involved in cell-cycle regulation and programmed cell death across multiple cancer types, including leukaemia [25], breast [26], lung [27], and gastrointestinal cancers [28].

Although capsaicin is widely recognized as a pungent irritant, recent preclinical work has demonstrated that its gastrointestinal effects are strongly dose-dependent. In mice, oral administration of 40 mg/kg capsaicin did not adversely affect gastrointestinal tissues, whereas higher doses (60–80 mg/kg) induced mucosal injury, intestinal inflammation, and elevations in serum neuropeptides such as substance P and CGRP [29]. Preclinical in vivo studies further demonstrate that capsaicin, administered orally in a dose of 20 mg/kg, reduces the tumour burden in colorectal cancer [30]. Because this study used oral administration, the observed antitumor effects may depend on local gastrointestinal exposure rather than systemic plasma levels. In this context, higher local exposure of capsaicin in the colon could be achieved through targeted drug-delivery systems, specifically designed to minimize its irritant effects in the upper gastrointestinal tract. Colon-specific formulations such as pH-dependent enteric coatings, time-controlled release capsules, and microbially triggered polysaccharide matrices can transport the compound through the upper gastrointestinal tract and release it only upon reaching the distal intestine [31]. Nanocarrier systems, including PLGA- or chitosan-based nanoparticles, can further enhance stability during transit and enable controlled release in response to colonic pH or bacterial enzymes [31]. These formulation strategies illustrate how localized delivery to the colon can be optimized, supporting the relevance of our in vitro model as a representation of targeted colonic exposure.

The concentration of capsaicin used in this study (200 µM, equivalent to 61.08 µg/mL) exceeds the highest serum levels reported in rat pharmacokinetic studies, in which an oral dose of 2 mg produced a Cmax of approximately 1.20 µg/mL after 90 min [32]. Although capsaicin shows a measurable tissue accumulation in vivo, as concentrations of 2.46 ± 0.07 ng/mL in the liver and 4.94 ± 0.26 ng/mL in the lung have been reported after 1 h of oral administration (10 mg/kg) in nude mice compared with 1.17 ± 0.01 ng/mL in plasma [33], no pharmacokinetic study has demonstrated capsaicin levels approaching 200 µM in the gut lumen or tumor microenvironment. Therefore, our use of this concentration should be interpreted as a mechanistic model of high local exposure rather than a reflection of clinically attainable tissue levels.

This study employed the Caco-2 colorectal cancer cell line to evaluate the cytotoxic effects of capsaicin and to examine its impact on the autophagy pathway and migratory capacity. For that, Caco-2 Cells were exposed to capsaicin across a range of concentrations (50–200 µM) for 24–72 h to assess its growth-inhibitory properties and to characterize associated molecular responses.

After 72 h of treatment, 200 µM capsaicin reduced cell growth to 54.82 ± 2.90% relative to the control group, indicating a pronounced cytotoxic effect on this cell line. This reduction in cell growth reflects both decreased cell viability and impaired proliferative capacity. Consistent with this observation, MTT and trypan blue exclusion assays demonstrated reduced metabolic activity and increased loss of membrane integrity, while SRB and wound-closure assays confirmed a significant inhibition of proliferative activity. Collectively, these findings indicate that capsaicin exerts both cytotoxic and antiproliferative effects in this model. 

The relatively low TRPV1 expression reported in Caco-2 cells may contribute to the reduced sensitivity of this model to capsaicin; however, this possibility was not directly tested in the present study. It is also important to note that capsaicin may exert cellular effects independently of TRPV1, as it can directly influence membrane fluidity, modulate ion fluxes, and alter intracellular ROS levels [34]. These TRPV1-independent mechanisms provide an additional explanation for the cellular responses observed at higher concentrations.

Because capsaicin at concentrations ≥100 µM disrupts epithelial barrier integrity and at 200–300 µM damages differentiated Caco-2 monolayers [35], these findings suggest that high luminal exposure may be deleterious to normal intestinal mucosa. Therefore, targeted delivery strategies capable of concentrating capsaicin at the tumour site while minimizing exposure to healthy epithelium may be required to safely exploit its anticancer potential.

The cell cycle is a highly organised series of events that culminate in cell division. This process is essential for cell maintenance and growth. Defects in the normal progression of the cell cycle, typically occurring at key regulatory checkpoints such as G1/S or G2/M, are defined as cell cycle arrest. During this interruption, the cell halts its advance toward division in response to DNA damage, cellular stress, or other harmful stimuli [36]. In this study, capsaicin successfully acted as a cell-cycle inhibitor that slowed and stopped proliferation by interfering with both DNA synthesis and cell division by blocking cells from entering mitosis (G2/M arrest) and simultaneously reducing DNA replication (reduced S-phase). These observations were accompanied by p21 upregulation and reduced phospho-p53 levels, suggesting that the observed G2/M arrest may involve altered regulation of p21 without activation of the canonical p53 phosphorylation response. Thus, the observed cell-cycle alterations may contribute to the reduced proliferative capacity of capsaicin-treated cells.

Evidence indicates that capsaicin-induced disruption of mitochondrial membrane potential and enhanced mitochondrial permeability can engage intracellular stress signaling, a TRPV1-independent stress response that can activate p21-mediated G2/M checkpoint engagement [37].

It is noteworthy that, according to our results, capsaicin did not activate apoptosis as a main mechanism of death, as previously detected in other cancer cell lines [38]. Neither cleaved PARP nor cleaved caspase-3 were not detected. Moreover, there was a reduction in the Sub-G0/G1 population, without a change in the nuclear morphology in cell cycle analysis and DAPI staining, respectively, suggesting the absence of apoptotic DNA fragmentation.

Because all analyses were performed after 72 h of treatment, without assessment of the initiator caspases-8 or -9, the data support the conclusion that apoptosis was not activated under the experimental conditions examined.

These observations are consistent with the findings of Chia-Han Lin et al., who reported that treatment of KB oral cancer cells with 250 µM capsaicin for 72 h resulted in reduced cell proliferation, attributable to a pronounced G2/M-phase arrest [39]. In HCT116 and LoVo colorectal cancer cells, capsaicin has been shown to induce p53-dependent G0/G1 arrest and apoptosis when applied at concentrations ranging from 80 to 300 µM for 24 h [14]. Furthermore, capsaicin induced ROS generation and a disruption of the mitochondrial transmembrane potential in Colo320DM colorectal cancer cells, resulting in DNA fragmentation, phosphatidylserine translocation, and a dramatic increase in caspase-3 activity [40]. In HT-29 human colon cancer cells, capsaicin induces apoptosis by activation of the peroxisome proliferator-activated receptor gamma [41]. In the same context, 200 µM of capsaicin inhibited cell proliferation of SW480 colorectal cancer cells by inducing cell cycle arrest in G_2_/M phase and activating the apoptosis pathway [42]. The differential responses observed in Caco-2 cells relative to other cancer cell lines exposed to capsaicin studied earlier reflect intrinsic differences, underscoring that the cellular response to capsaicin is highly cell-type dependent.

Under stress conditions, cells sense harmful marks and respond by activating survival pathways, such as the autophagy pathway. Autophagy allows cells to adapt to harmful conditions by forming an autophagosome that will later fuse with lysosomes, forming autolysosomes, allowing the degradation of damaged components, restoring homeostasis, and maintaining survival. Any disruption in these sequential steps compromises the autophagic pathway, leading to impaired cargo degradation and the intracellular accumulation of potentially harmful components [43].

In this model, capsaicin treatment resulted in an accumulation of the LC3B-II/LC3B-I ratio, a pattern indicative of either enhanced autophagosome formation (active flux) or impaired autophagic flux. Concurrently, capsaicin impaired lysosomal degradative capacity by reducing the level of mature cathepsin-D, a key lysosomal protease required for autophagic cargo breakdown. Although pro-cathepsin-D abundance remained comparable between control and treated cells, the marked reduction in the mature form suggests an alteration in cathepsin-D processing or stability, rather than a simple loss of total cathepsin-D expression. Additional studies are therefore required to elucidate the underlying mechanism, particularly to determine whether capsaicin interferes with post-translational maturation, lysosomal trafficking, or the stability of cathepsin-D within the acidic lysosomal environment. Although our results show a positive correlation between mature cathepsin-D protein levels and viability, a causal effect would require additional experiments, such as cathepsin-D overexpression or knockdown followed by viability assessment after capsaicin treatment, to confirm the protective effect of cathepsin-D on Caco-2 cell viability.

It is important to mention that capsaicin can reduce mature cathepsin-D through several TRPV1-independent mechanisms, including mitochondrial injury and ROS generation, which are well-known to impair lysosomal acidification and protease processing, thereby preventing the conversion of pro-cathepsin-D into its mature, active form. Additionally, capsaicin-induced alterations in membrane fluidity can disrupt vesicular trafficking and lysosomal stability, further contributing to reduced cathepsin-D maturation [34].

This impairment resulted in LC3B-II accumulation without affecting lysosomal biogenesis or autophagosome–lysosome fusion, as evidenced by unchanged LAMP-1 expression and preserved LC3B–LAMP-1 co-localisation. Moreover, CQ was employed as a lysosomal inhibitor to assess autophagic flux. Results showed that co-treatment with capsaicin and CQ failed to further increase p62 or LC3B-II levels, indicating that capsaicin had already imposed an impairment on autophagic degradation, rendering additional lysosomal inhibition by CQ ineffective. The positive association between cathepsin-D expression and cell viability, together with its negative association with the LC3B-II/LC3B-I ratio and p62 levels, indicates that reduced lysosomal proteolytic capacity—and consequent impairment of autophagic cargo degradation—is linked to the cytotoxic effects of capsaicin in this model. In a previous study conducted on human oral squamous carcinoma cell lines (HSC-3 and SAS), treatment with 200 µM capsaicin for 48 h induced autophagic flux without activating apoptosis, further supporting the notion that the cellular response to capsaicin is highly cell-line dependent [44].

Additionally, capsaicin exerted a clear anti-migratory effect in Caco-2 cells, as evidenced by the reduced wound-closure rate in CAP-treated cultures relative to the control group. This diminished migratory capacity is consistent with the observed upregulation of occludin, a key tight-junction protein whose increased expression is associated with strengthened cell–cell adhesion and restricted epithelial cell motility. These findings suggest that occludin may contribute to the reduced migratory capacity observed in this model; however, additional experiments, such as occludin knockdown, are required to confirm a direct causal relationship. Furthermore, the use of cytosine arabinoside without assessing its potential effects on cytoskeletal dynamics represents a limitation of the present study. This result is consistent with previous findings reporting occludin overexpression under cellular stress conditions, which was associated with a reduction in migratory capacity [45].

In parallel, capsaicin can enhance tight-junction integrity independently of TRPV1 by suppressing LPS–TLR4–NF-κB inflammatory signaling. Studies using TRPV1-knockout mice and TRPV1-/- Caco-2 cells demonstrated that capsaicin reduces intestinal LPS levels, downregulates TLR4, and lowers TNF-α and IL-6 expression. This anti-inflammatory shift promotes tight-junction restoration [46].

This study presents some limitations that should be acknowledged. First, theconcentrations of capsaicin required to induce cytotoxic effects in Caco-2 cells exceed currently achievable plasma levels in vivo, supporting the exploratory nature of this in vitro investigation. Second, the present study was performed in a single colorectal cancer cell line. Because cellular responses to capsaicin may vary substantially according to the molecular characteristics and TRPV1 expression profile of individual cell lines, the findings obtained in Caco-2 cells cannot be assumed to represent a general mechanism in colorectal cancer. Therefore, the main observations reported here, including autophagy impairment, cathepsin-D downregulation, and G2/M cell-cycle arrest, will need to be reproduced and further investigated in additional CRC models, such as HCT116 and SW480, which display different molecular and TRPV1 expression profiles. Such comparative studies will be important to determine whether the observed effects are conserved across CRC models or are instead specific to the Caco-2 cellular context, including its relatively low basal TRPV1 expression. They may also clarify whether the higher capsaicin concentrations required in our experiments are related to cell-line-specific receptor abundance or other cellular characteristics.Thus, the present findings should be regarded as exploratory and hypothesis-generating, providing a preliminary framework for further investigation rather than establishing a definitive mechanism of capsaicin action in colorectal cancer.

## 4. Materials and Methods

### 4.1. Cell Culture

The human colon adenocarcinoma Caco-2 cell line is widely used as an in vitro model of colorectal cancer owing to its origin from human colon carcinoma and its capacity to recapitulate several phenotypic features of malignant intestinal epithelial cells [47].

Caco-2 cells at passages 45–50 (American Type Culture Collection, ATCC, Manassas, VA, USA) were employed for all experiments. Cells were maintained in high-glucose Dulbecco’s Modified Eagle Medium (DMEM) supplemented with 10% fetal bovine serum (FBS), 1% L-glutamine, and 100 U/mL penicillin/streptomycin (Euroclone S.p.A., Milan, Italy). Cultures were grown in T75 flasks at 37 °C in a humidified atmosphere containing 5% CO_2_, with medium renewal every 48 h. Cells were passaged upon reaching approximately 80% confluence. For starvation experiments, Caco-2 cells were seeded as described above and subjected to nutrient deprivation using low-glucose DMEM lacking both glutamine and FBS for 6 h prior to the treatment endpoint [48].

To verify autophagic flux, chloroquine (QC), as a late-stage lysosomal inhibitor [49], was added at a final concentration of 50 µM or 75 µM for 6 h prior to the treatment endpoint. The used concentrations were not able to induce excessive cytotoxicity according to a previously published work [50].

### 4.2. Capsaicin Preparation

A stock solution of capsaicin (0.16 M) was prepared by dissolving the capsaicin powder (Sigma-Aldrich, St. Louis, MO, USA) in sterilized ethanol (Sigma-Aldrich, St. Louis, MO, USA). Consequently, working concentrations of capsaicin (50, 100, and 200 µM) were prepared by diluting the stock solution in DMEM. The ethanol group (vehicle) was included to account for any potential effects of the used ethanol concentration (0.125% *v*/*v*) on cell viability.

### 4.3. MTT Assay

Cell viability was evaluated using the standard MTT assay [51]. Caco-2 cells were seeded at 1 × 10^4^ cells/well in 96-well plates and allowed to adhere for 24 h before treatment. Cells were then exposed to the specified concentrations of capsaicin for 24, 48, or 72 h. Following treatment, the medium was replaced with 200 µL of MTT solution (0.5 mg/mL) and incubated for 4 h at 37 °C in 5% CO_2_. The resulting formazan crystals were solubilized in 150 µL of DMSO (Sigma-Aldrich), and absorbance was recorded at 650 nm using a microplate reader.

### 4.4. Sulforhodamine B (SRB) Assay

The SRB assay was employed to evaluate the cytotoxic effects of capsaicin on Caco-2 cell growth [52]. Caco-2 cells were seeded at 1 × 10^4^ cells/well in 96-well plates and incubated overnight. The next day, Cells were treated with 200 µM capsaicin for 24, 48, or 72 h. After treatment, cells were fixed with cold 50% (*v*/*v*) trichloroacetic acid for 1 h at 4 °C, washed four times with distilled water, and air-dried. A 0.04% (*w*/*v*) SRB solution was added and incubated for 1 h at room temperature. Unbound dye was removed by washing four times with 1% (*v*/*v*) acetic acid. After drying, the protein-bound dye was solubilized in 50 µL of 10 mM Tris base, and fluorescence was measured at 488 nm excitation and 560 nm emission using a microplate reader.

### 4.5. Cell Cycle Analysis

Cell-cycle analysis was performed by flow cytometry following propidium iodide staining. Caco-2 cells (5 × 10^5^) were seeded in 6-well plates in complete medium containing 10% FBS and allowed to adhere overnight. Cells were then treated with 200 µM capsaicin or maintained in control medium for 72 h. After treatment, cells were washed three times with PBS, trypsinized, and collected by centrifugation at 300× *g* for 5 min. Pellets were fixed in 100% ethanol at −20 °C overnight, washed twice with PBS, and incubated in 0.5 mL PBS containing RNase A (100 µg/mL; R6148, Sigma-Aldrich) and propidium iodide (PI, 50 µg/mL; P4170, Sigma-Aldrich) at 37 °C for 30 min. Samples were analyzed with a FACSCalibur™ cytofluorimeter using CELLQuest software version 3.3 (BD Biosciences, Franklin Lakes, NJ, USA). PI excitation was performed with an argon-ion laser at 488 nm, and PI emission was recorded with the FL2 channel (filter 585/42 nm, detection range 564–606 nm). Forward scatter (FS) and side scatter (SS) were used to identify single cells, and pulse processing was used to exclude cell doublets from the analysis (pulse area vs. pulse width). For analysis, a gate was drawn around the single-cell population using pulse width versus pulse area, and this gate was then applied to the scatter plot to exclude obvious debris. The resulting combined gates were then applied to the PI histogram plot. A total of 10,000 events per sample were acquired.

### 4.6. Trypan Blue Exclusion Assay

Cell viability was also assessed using the Trypan Blue exclusion assay. Caco-2 cells were seeded at 2 × 10^5^ cells per well in six-well plates and exposed either to 200 µM capsaicin or to standard culture medium for 72 h. After treatment, cells were washed with PBS, detached using Trypsin-EDTA, centrifuged, and resuspended to obtain a uniform cell suspension. An equal volume of 0.4% Trypan Blue solution was added, and the mixture was gently homogenized. Following a 3 min incubation at room temperature, the stained suspension was loaded onto a hemocytometer. Viable cells remained unstained, whereas non-viable cells exhibited blue staining. Cell viability was expressed as the percentage of unstained cells relative to the total number of cells counted.

### 4.7. Scratch Assay

The scratch assay primarily measures cell migration, although cell proliferation may also contribute to wound closure. This assay was performed following the protocol described by Liang et al. (2007) [53]. Caco-2 cells were seeded at 1.5 × 10^5^ cells/well in 24-well plates and allowed to reach confluence (approximately 24 h). A linear scratch was generated across the monolayer using a sterile pipette tip, and images of the wound area were captured at 0, 24, 48, and 72 h using a LEICA DMI 4000 B phase-contrast microscope (Leica Microsystems, Wetzlar, Germany). Wound closure was quantified by measuring the change in wound area over time, and the percentage of closure was calculated as:

Wound closure (%) = 100 × (Initial area (t_0_) − Final area (t))/Initial area (t_0_)

To account for differences in cell density and proliferative capacity, SRB assays were performed in parallel under identical conditions, and wound-closure values were normalized to the corresponding SRB signal:

Normalized wound closure (%) = Wound closure (%)/SRB signal

To distinguish migration-dependent from proliferation-dependent effects, two assay conditions were employed. For the migration-focused assay, Caco-2 cells were pre-screened with cytosine arabinoside (Ara-C; Sigma-Aldrich, Cat. No. C1768) to identify a non-toxic concentration capable of suppressing proliferation. Based on this screening, 10 µM Ara-C was added to both control and capsaicin-treated groups 2 h before scratch formation and maintained throughout the assay. For the proliferation-inclusive assay, Ara-C was omitted, allowing wound closure to reflect both migration and proliferation. The migration rate was calculated as:

Migration rate (µm/h) = (Distance(t) − Distance(t_0_))/Time elapsed (h)

### 4.8. Western Blot Analysis

Caco-2 cells treated with 200 µM capsaicin for 72 h were lysed in RIPA buffer supplemented with protease and phosphatase inhibitors (Sigma-Aldrich). Cells were scraped, incubated on ice for 15 min, sonicated, and centrifuged at 13,000× *g* for 5 min at 4 °C. Protein concentrations were determined using the Bradford assay (Bio-Rad Laboratories, Hercules, CA, USA) [30]. A total of 40 µg of protein was separated by SDS-PAGE and transferred to nitrocellulose membranes. Membranes were then stained with Ponceau S to verify protein transfer, blocked with 5% milk in TBST (0.1% Tween-20) for 1 h, and incubated overnight at 4 °C with primary antibodies against LC3B (Cell Signaling Technology, Danvers, MA, USA; Cat. No. 43566S, Lot No. 14; dilution 1:1000), cathepsin-D (Cell Signaling Technology, Danvers, MA, USA; Cat. No. 2284S, Lot No. 2; dilution 1:1000), occludin (Cell Signaling Technology, Danvers, MA, USA; Cat. No. 91131S, Lot No. 3; dilution 1:1000), PARP (Cell Signaling Technology, Danvers, MA, USA; Cat. No. 9542S, Lot No. 15; dilution 1:1000), cleaved caspase-3 (Cell Signaling Technology, Danvers, MA, USA; Cat. No. 9661S, Lot No. 43; dilution 1:1000), p21 (Cell Signaling Technology, Danvers, MA, USA; Cat. No. 2947S, Lot No. 15; dilution 1:1000), p62 (Cell Signaling Technology, Danvers, MA, USA; Cat. No. 5114S, Lot No. 4; dilution 1:1000), and LAMP-1 (Santa Cruz Biotechnology, Dallas, TX, USA; Cat. No. 9091S, Lot No. G1420; dilution 1:1000). Finally, membranes were washed and incubated with secondary antibodies (Goat anti-Rabbit and Goat anti-Mouse; Bio-Rad; dilution 1:5000) for 1 h at room temperature. Protein bands were visualized using the Clarity Western ECL Substrate kit (Bio-Rad Laboratories, Hercules, CA, USA) and normalized to housekeeping proteins.

### 4.9. Immunofluorescence Staining

Caco-2 cells were seeded at 2 × 10^5^ cells on glass coverslips placed in six-well plates and treated as described. Cells were fixed with 4% paraformaldehyde and permeabilized with 0.2% Triton X-100 in PBS (Sigma-Aldrich) to enable antibody access to intracellular compartments. After PBS washing, nonspecific binding was blocked with 1% bovine serum albumin (BSA) for 1 h at room temperature. Cells were then incubated overnight at 4 °C with primary antibodies against LC3B (1:100 in PBS) and LAMP-1 (1:100 in PBS). Following washing and gentle drying, fluorophore-conjugated secondary antibodies—Alexa Fluor 488 (Invitrogen, Carlsbad, CA, USA; Lot 1797971) and Alexa Fluor 555 (Invitrogen, Carlsbad, CA, USA; Lot 2214478)—were applied at a 1:1000 dilution in PBS for 1 h at room temperature. Samples were washed, dried, and mounted using antifade medium. Confocal images were acquired using sequential scanning to minimize spectral bleed-through, with laser power and detector settings optimized to avoid signal saturation. High-resolution images were obtained using a 63× objective. Image analysis was performed using ImageJ/Fiji (version 1.54r). To minimize variability arising from differences in cell density, entire microscopic fields containing at least 10 cells were selected based on brightfield images. Brightfield images were used to verify cell distribution and exclude fields with overlapping or sparsely populated cells. Nine fields per condition were analyzed across three independent experiments (three fields per condition per experiment), with each field containing at least 10 cells. For each field, a single region of interest (ROI) encompassing the entire field was defined for quantitative analysis. Background fluorescence was subtracted using a cell-free region of the corresponding image, followed by thresholding and quantification of fluorescence co-localization using Manders’ coefficients. Thus, nine ROIs per condition were analyzed across the three independent experiments.

### 4.10. DAPI-Based Nuclear Staining

Caco-2 cells were seeded at 2 × 10^5^ cells on glass coverslips in six-well plates and treated with capsaicin or standard medium for 72 h. Cells were fixed with 4% paraformaldehyde in PBS for 10–15 min at room temperature and washed three times with PBS. Fixed cells were incubated with DAPI (4′,6-diamidino-2-phenylindole) at a final concentration of 1 µg/mL in PBS for 5–10 min at room temperature in the dark. After staining, cells were rinsed with PBS and mounted in aqueous medium. Fluorescence images were acquired using Axio Vision (Carl Zeiss, Oberkochen, Germany) and LEICA DMRB (Leica Microsystems, Wetzlar, Germany) microscopes equipped with a DAPI filter set (excitation ~358 nm, emission ~461 nm). Overview images were captured with a 10× objective, and high-magnification images with a 63× objective. Exposure time, gain, and all acquisition parameters were kept constant within each experiment to ensure comparability across samples.

### 4.11. Transmission Electron Microscopy (TEM) Images

Caco-2 cells were seeded at 2 × 10^5^ cells in six-well plates and treated with capsaicin or standard medium for 72 h. Cells were fixed in 2.5% glutaraldehyde in 0.1 M cacodylate buffer (pH 7.4) for 2 h, washed in buffer, and post-fixed in 1.5% potassium ferrocyanide and 1% osmium tetroxide for 1 h. Samples were rinsed with water and stained en bloc in 0.5% aqueous uranyl acetate overnight. After dehydration through a graded ethanol series, samples were embedded in epoxy resin and polymerized at 60 °C. Ultrathin sections (70 nm) were cut using a Reichert Ultracut E ultramicrotome (Wetzlar, Germany) and collected on 100-mesh copper grids. Sections were examined using a Tecnai G2 Spirit transmission electron microscope (FEI Company, Hillsboro, OR, USA) operated at 120 kV and equipped with a TVIPS F-216 CMOS camera for digital image acquisition. Specifically, autophagosomes were identified based on their characteristic ultrastructural features, including membrane-bound vesicular structures containing cytoplasmic material and/or partially degraded cellular components. The quantification was performed by counting the number of autophagosomes per cell. For each experimental condition, three independent biological replicates were analyzed, with nine randomly selected microscopic fields evaluated per replicate. The analysis was performed on coded samples to minimize observer bias.

### 4.12. Data Analysis

The Shapiro–Wilk normality test was used to assess the normal distribution of the obtained data. Student’s *t*-test for independent samples was used to compare the mean of the studied parameters between two independent groups with normally distributed data. Post hoc multiple comparisons were performed using Dunnett’s correction to compare each treatment group with the control. Pearson’s correlation coefficient (r) was used to quantify the correlation between two continuous numerical variables. Effect sizes were quantified using Cohen’s d. Each experiment (n = 3) represents an independent biological replicate performed on separate cell cultures. Each condition was tested in multiple technical replicates within each experiment. The results were presented as the mean ± standard error of the mean (SEM). GraphPad Prism 6.0.1 and the Statistical Package for the Social Sciences (SPSS) version 20 were utilized for statistical analysis. A *p*-value of less than 0.05 was considered significant.

## 5. Conclusions

In summary, capsaicin significantly reduced Caco-2 cell viability and proliferation and induced G2/M cell-cycle arrest, accompanied by p21 upregulation and reduced p53 phosphorylation. Instead, capsaicin impaired autophagic degradation via cathepsin-D downregulation, leading to a late-stage blockade of autophagic flux that contributed to cytotoxicity. Capsaicin also suppressed cell migration, consistent with increased occludin protein expression and enhanced tight-junction integrity. Collectively, these results highlight impaired autophagic degradation, cell-cycle arrest, and reduced motility as key mechanisms underlying the anticancer effects of capsaicin in Caco-2 colon cancer cells.

## Figures and Tables

**Figure 1:**
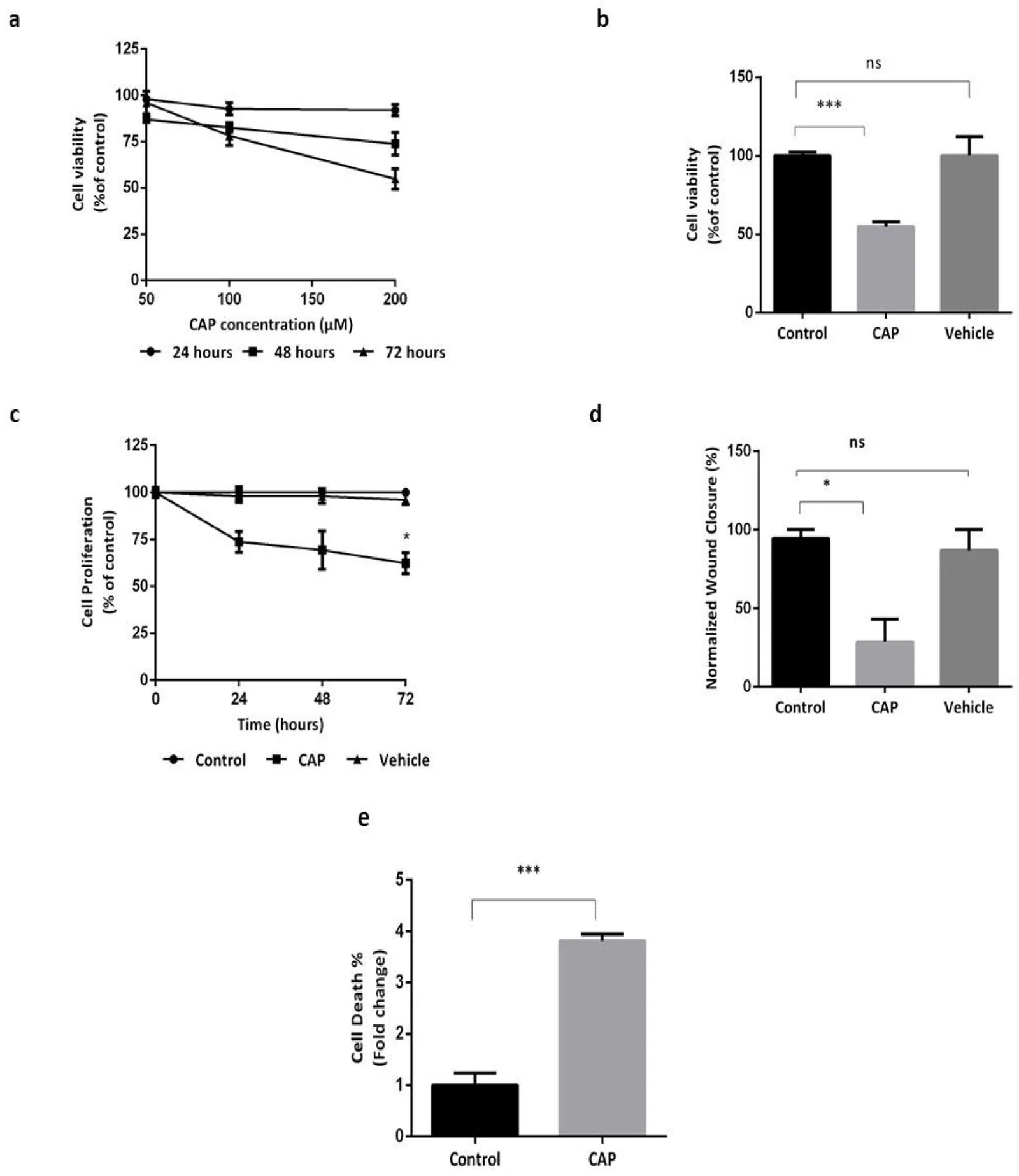
Capsaicin reduces the viability and growth of Caco-2 cells: (**a**) MTT assay results (50, 100, 200 µM for 24, 48, 72 h) show a time- and dose-dependent decline in cell viability in capsaicin treatment compared to the control group. The y-axis reflects percentage viability relative to untreated control; a decrease to ~55% indicates marked cytotoxicity. (**b**) At 200 µM for 72 h, capsaicin reduced viability compared to the control (*p* < 0.001), while the vehicle had no significant effect. (**c**) The SRB assay (treatment with 200 µM capsaicin for 24, 48, 72 h) indicates a significant inhibition of the proliferation rate over time, with cell density decreasing to 62.28 ± 5.60% of control at 72 h (*p* < 0.05). (**d**) The scratch assay (treatment with 200 µM capsaicin for 72 h) showed a significant decrease in the normalized wound closure in capsaicin-treated cells (28.54 ± 14.38%) compared to the control (100 ± 5.70%) after 72 h of treatment (*p* < 0.05). (**e**) Trypan blue exclusion analysis (treatment with 200 µM capsaicin for 72 h) exhibited a significant increase in non-viable cells following capsaicin treatment (*p* < 0.001). All values represent mean ± SEM (N = 3). Statistical significance: * *p* < 0.05, *** *p* < 0.001 compared to control, ns: not significant.

**Figure 2:**
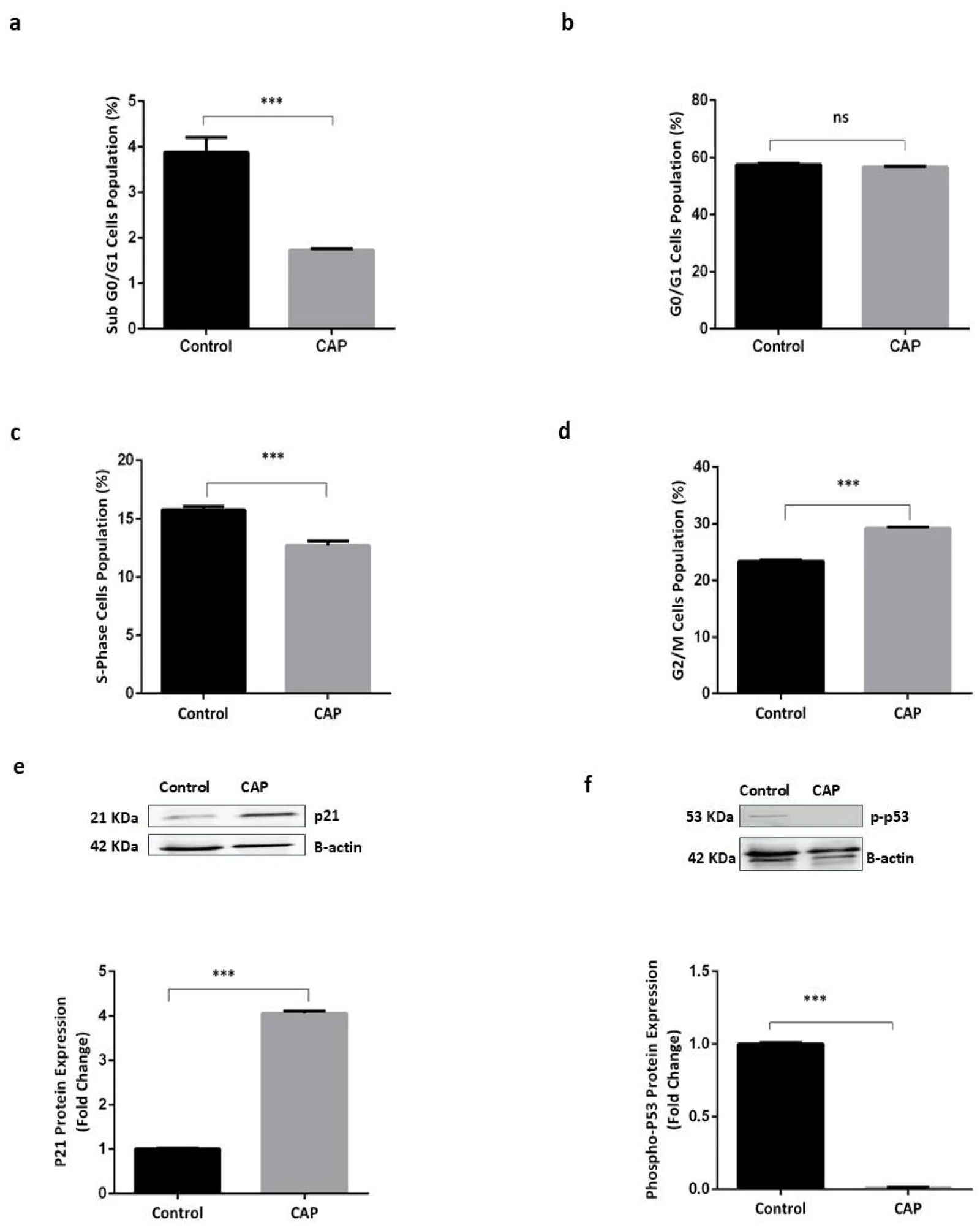
Capsaicin induces G2/M cell cycle arrest in Caco-2 cells without triggering apoptosis. (**a**): Flow cytometry analysis of DNA content after 72 h of treatment with 200 µM Capsaicin reduced the Sub-G0/G1 fraction (1.73 ± 0.03% vs. 3.88 ± 0.33%; *p* < 0.01), (**b**): unchanged in G0/G1 phase cells population was detected (*p* > 0.05), (**c**) while a significant decrease in S-phase was observed (12.70 ± 0.37% vs. 15.74 ± 0.32% *p* < 0.01). (**d**): Significant accumulation of G2/M was observed (29.19 ± 0.21 vs. 23.34 ± 0.22%; *p* < 0.001). (**e**,**f**): Capsaicin upregulates p21 expression (*p* < 0.05) and reduces phospho-p53 levels (*p* < 0.001), in association with G2/M cell-cycle arrest. All values represent mean ± SEM (N = 3). Statistical significance: ns: not significant; *** *p* < 0.001 compared to control.

**Figure 3:**
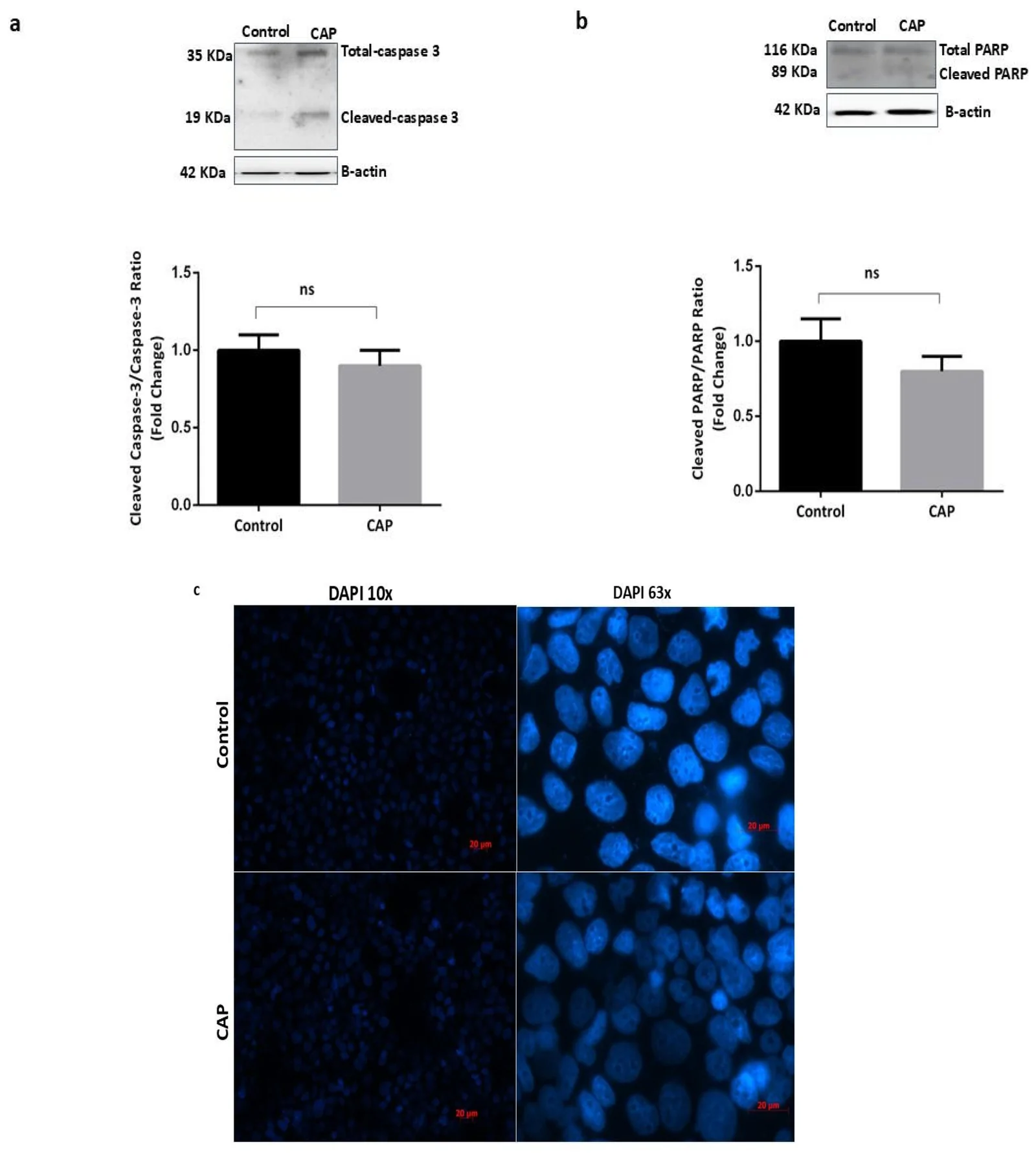
**Capsaicin does not induce caspase-dependent apoptosis:** (**a**,**b**) Cleaved/total PARP ratio and Cleaved Caspase-3/Caspase-3 ratio, in cells treated with 200 µM capsaicin for 72 h, remained unchanged across groups (*p* > 0.05). (**c**) DAPI staining of control and capsaicin-treated Caco-2 cells imaged at 10× and 63× magnification. Nuclei appeared round, intact, and uniformly stained in both groups, with no evidence of chromatin condensation, nuclear fragmentation, or other apoptotic-associated abnormalities. All values represent mean ± SEM (N = 3). Statistical significance: ns: not significant compared to control.

**Figure 4:**
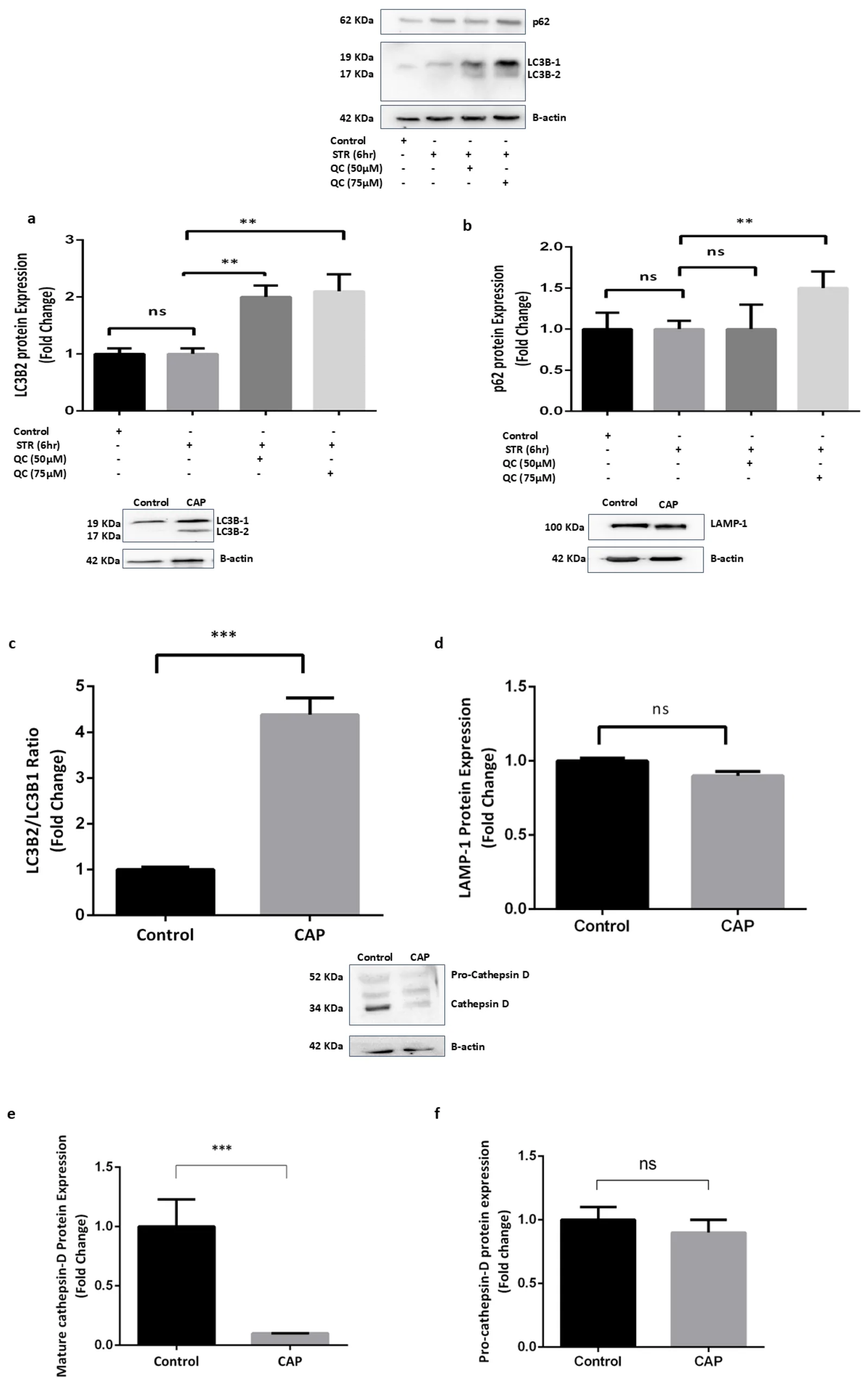
Capsaicin activates autophagosome formation but impairs lysosomal degradation in Caco-2 cells: (**a**) Serum starvation for 6 h did not significantly increase LC3B-II levels relative to control cells (*p* > 0.05), indicating efficient lysosomal degradation under basal conditions. Co-treatment of starved cells with 50 or 75 µM CQ induced a marked accumulation of LC3B-II (*p* < 0.01), confirming effective inhibition of lysosomal degradation in this model. (**b**) p62/SQSTM1 levels remained unchanged under starvation (*p* > 0.05), consistent with sufficient autophagic turnover. In contrast, 75 µM CQ induced significant p62 accumulation (*p* < 0.01), whereas 50 µM CQ had no significant effect (*p* > 0.05), reflecting partial lysosomal blockade at the lower concentration. (**c**) Capsaicin treatment significantly increased the LC3B-II/LC3B-I ratio (*p* < 0.01), indicating either enhanced autophagosome formation or impaired autophagosome turnover. (**d**) LAMP-1 expression remained unchanged (*p* > 0.05), suggesting preserved lysosomal abundance and intact autophagosome–lysosome fusion. (**e**,**f**) Treatment with 200 µM capsaicin for 72 h markedly reduced the mature-cathepsin-D level (*p* < 0.001), without a significant effect on pro-cathepsin-D level (*p* > 0.05), demonstrating impaired lysosomal proteolytic capacity and supporting a defect in autophagic degradation rather than lysosomal biogenesis. Statistical significance: ns: not significant; ** *p* < 0.01 compared to control, *** *p* < 0.001 compared to control.

**Figure 5:**
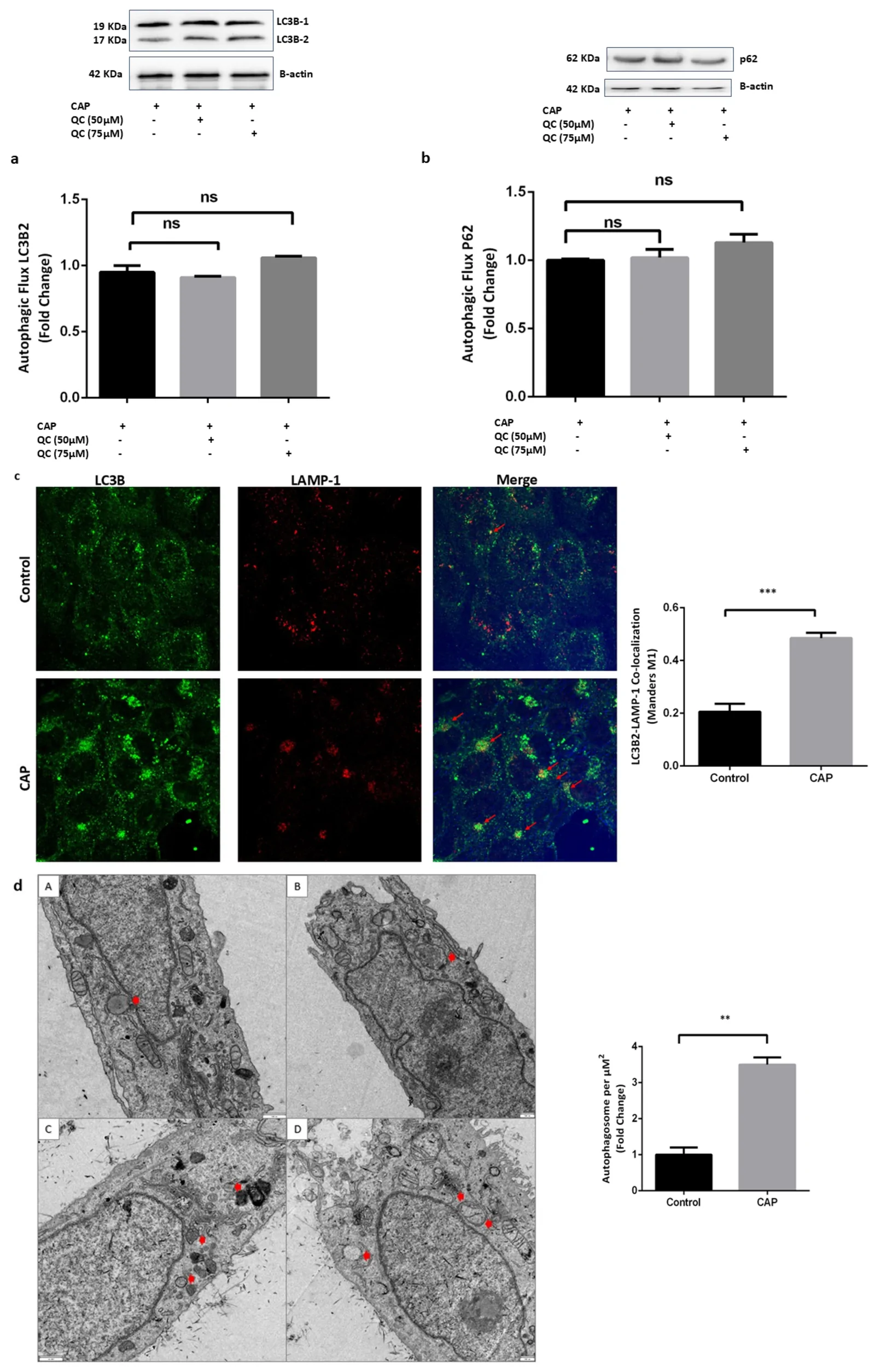
**Capsaicin impairs autophagic degradation despite preserved autophagosome–lysosome interactions in Caco-2 cells:** (**a**,**b**) Autophagic flux assessment in cells treated with 200 µM capsaicin for 72 h, co-treated with 50 or 75 µM CQ. CQ failed to induce additional accumulation of LC3B-II or p62 at either 50 or 75 µM (*p* > 0.05), indicating that autophagic degradation was already maximally impaired prior to lysosomal inhibition. (**c**) Confocal microscopy revealed a significant increase in LC3B-II/LAMP-1 co-localization in capsaicin-treated cells compared with controls (*p* < 0.001), demonstrating enhanced autophagosome–lysosome interactions. (**d**) Transmission electron microscopy showed a pronounced accumulation of double-membrane autophagosomes in capsaicin-treated cells (panels C & D) relative to untreated controls (panels A & B), as autophagosome formation increases significantly in capsaicin-treated cells (*p* < 0.01). Panels (A, B, C, D) include a 500 nm scale bar. Data are presented as mean ± SEM (N = 3). Statistical significance: ns: not significant; ** *p* < 0.01 vs. control, *** *p* < 0.001 vs. control.

**Figure 6:**
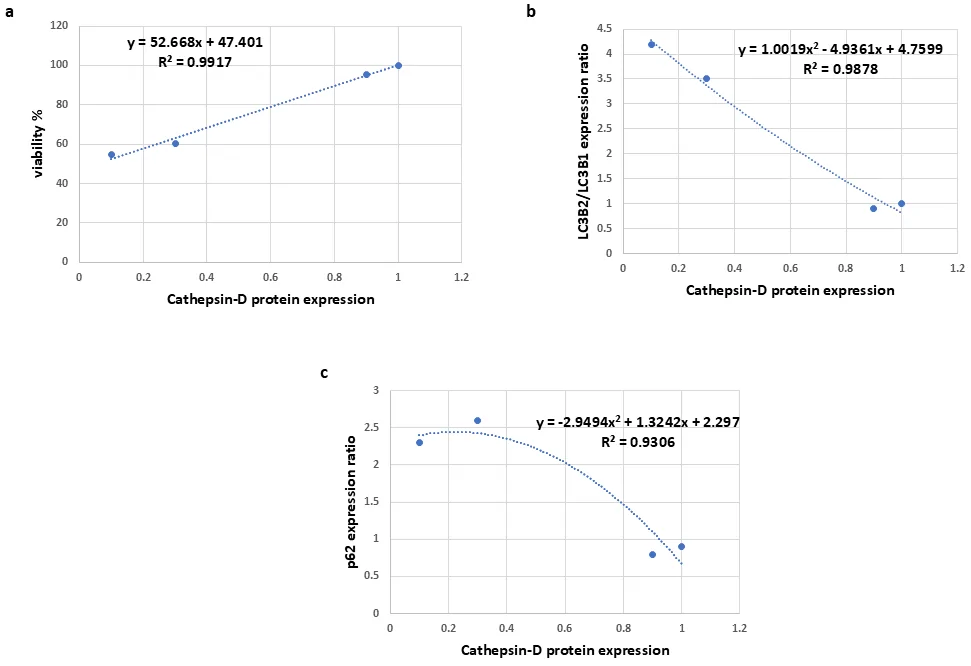
**Cathepsin-D expression correlates with cell viability and markers of impaired autophagic degradation in Caco-2 cells:** (**a**) Correlation analysis, in cells treated with 200 µM capsaicin for 72 h, between cathepsin-D protein expression and cell viability showed a strong positive association (Pearson r = 0.99, *p* < 0.01), suggesting that cathepsin-D downregulation is associated with reduced cell viability. (**b**,**c**) Reduced cathepsin-D expression correlated negatively with LC3B-II levels (Pearson r = −0.96, *p* < 0.01) and p62/SQSTM1 accumulation (Pearson r = −0.91, *p* < 0.01). All experiments were performed using four independent biological replicates.

**Figure 7:**
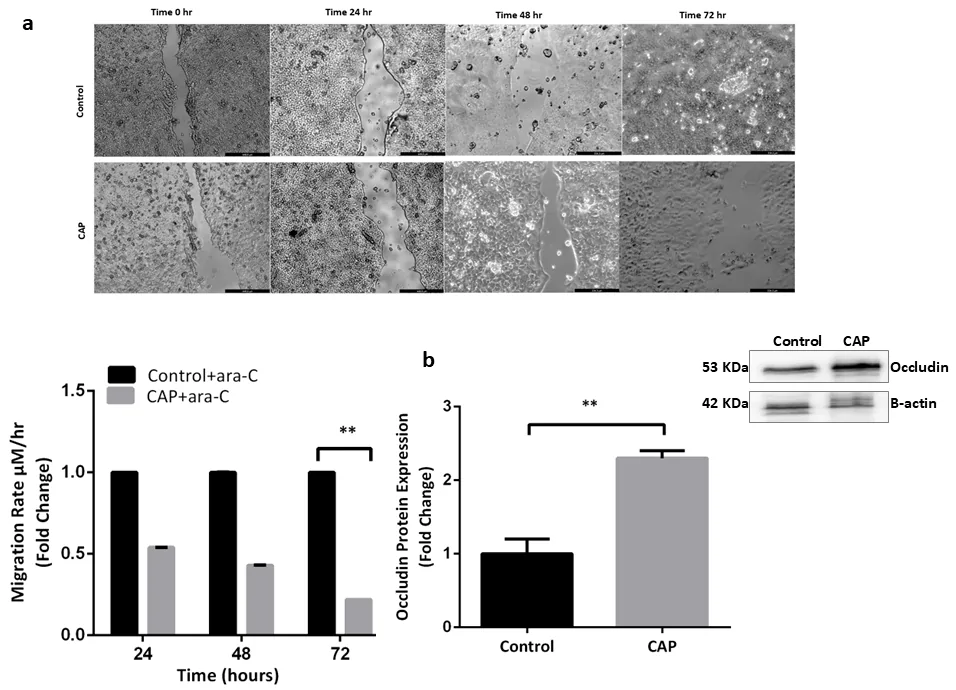
**Capsaicin reduces migration rate and increases occludin expression in Caco-2 cells:** (**a**) Treatment with 200 µM capsaicin for 72 h in the presence of ara-C reduced wound closure to 0.22-fold relative to ara-C controls (*p* < 0.01). (**b**) Treatment with 200 µM capsaicin for 72 h significantly increased occludin expression (*p* < 0.01), indicating strengthened tight-junction integrity and supporting reduced motility. Data are presented as mean ± SEM (N = 3). Statistical significance: ** *p* < 0.01 vs. control.

## Data Availability

All data and analysis are available within the manuscript or upon request to the corresponding authors.

## Abbreviations

The following abbreviations are used in this manuscript:

ACD	Autophagic Cell Death
ATCC	American Type Culture Collection
AIF	Apoptosis-Inducing Factor
CRC	Colorectal Cancer
CAP	Capsaicin
CO_2_	Carbon Dioxide
DMSO	Dimethyl Sulfoxide
DMEM	Dulbecco’s Modified Eagle Medium
ECL	Enhanced Chemiluminescence
ER	Endoplasmic Reticulum
PC12	Pheochromocytoma Cells
FBS	Fetal Bovine Serum
LC3B	Microtubule-Associated Protein 1 Light Chain 3 Beta
mTOR	Mammalian Target Of Rapamycin
MTT	3-(4,5-Dimethylthiazol-2-Yl)-2,5- Diphenyltetrazolium Bromide
OD	Optical Density
PARP	Poly (ADP-Ribose) Polymerase
PBS	Phosphate-Buffered Saline
PI3K	Phosphoinositide 3-Kinase
RIPA	Radioimmunoprecipitation Assay
ROS	Reactive Oxygen Species
SDS-PAGE	Sodium Dodecyl Sulfate–Polyacrylamide Gel Electrophoresis
SRB	Sulforhodamine B
TBS	Tris-Buffered Saline
TBST	TBS with Tween 20
TCA	Trichloroacetic Acid
WHO	World Health Organization
